# Supplementary material for: The distribution of registered occupational therapists, physiotherapists, and podiatrists in Australia
Source: PLoS One. 2023 Sep 21;18(9):e0291962. doi: 10.1371/journal.pone.0291962 (PMC10513188; doi:10.1371/journal.pone.0291962)
Supplement: S1 Table — (PDF) [file pone.0291962.s001.pdf]

S1 Table. Proportion of registered occupational therapists, physiotherapists, and podiatrists by MMM in Australia and in each state and territory, April 2020

Australia

MMM2019	Profession<br>Occupational Therapist (n=23248)	Physiotherapist (n=33828)	Podiatrist (n=5512)	Usual resident Population
1	18061 (77.7%)	27491 (81.3%)	4228 (76.7%)	16,633,903
2	2270 (9.8%)	2527 (7.5%)	496 (9.0%)	2,106,918
3	1513 (6.5%)	1880 (5.6%)	414 (7.5%)	1518211
4	656 (2.8%)	929 (2.7%)	190 (3.4%)	929192
5	541 (2.3%)	733 (2.2%)	132 (2.4%)	1,690,161
6	155 (0.7%)	210 (0.6%)	40 (0.7%)	274908
7	52 (0.2%)	58 (0.2%)	12 (0.2%)	201826

NB: The total population excludes residents living in SA1 areas (33 SA1 areas) that do not classify by MMM (n=46,342)

New South Wales

MMM2019	Profession<br>Occupational Therapist (n= 6548)	Physiotherapist (n= 10228)	Podiatrist (n=1548)	Usual resident Population
1	5164 (78.9%)	8405 (82.2%)	1230 (79.5%)	5577427
2	211 (3.2%)	203 (2.0%)	40 (2.6%)	180452
3	774 (11.8%)	1006 (9.8%)	183 (11.8%)	733203
4	208 (3.2%)	340 (3.3%)	52 (3.4%)	378029
5	188 (2.9%)	263 (2.6%)	39 (2.5%)	563174
6	3 (<1%)	11 (0.1%)	4 (0.3%)	29644
7	0	0	0	5665

Victoria

MMM2019	Profession<br>Occupational Therapist (n=6053)	Physiotherapist (n=8721)	Podiatrist (n=1767)	Usual resident Population
1	4816 (79.6%)	7316 (83.9%)	1338 (75.7%)	4556221
2	506 (8.4%)	531 (6.1%)	163 (9.2%)	402965
3	334 (5.5%)	369 (4.2%)	124 (7.0%)	299736
4	239 (3.9%)	316 (3.6%)	91 (5.1%)	265258
5	158 (2.6%)	187 (2.1%)	51 (2.9%)	391502
6	0 (0.0%)	2 (<1%)	0 (0.0%)	3176

Queensland

MMM2019	Occupational Therapist (n=4706)	Physiotherapist (n=6675)	Podiatrist (n=981)	Usual resident Population
1	3360 (71.4%)	5195 (77.8%)	741 (75.5%)	2957153

2	1016 (21.6%)	1075 (16.1%)	171 (17.4%)	922312
3	72 (1.5%)	88 (1.3%)	17 (1.7%)	135043
4	137 (2.9%)	157 (2.4%)	23 (2.3%)	199764
5	67 (1.4%)	98 (1.5%)	10 (1.0%)	350727
6	34 (0.7%)	46 (0.7%)	11 (1.1%)	68008
7	20 (0.4%)	16 (0.2%)	8 (0.8%)	57465

South Australia

**MMM2019**	**Profession**			**Usual resident Population**
	**Occupational Therapist (n=1810)**	**Physiotherapist (n=2763)**	**Podiatrist (n=504)**	
1	1557 (86.0%)	2374 (85.9%)	422 (83.7%)	1230258
2	9 (0.5%)	21 (0.8%)	2 (0.4%)	34172
3	115 (6.4%)	153 (5.5%)	32 (6.3%)	133470
4	47 (2.6%)	74 (2.7%)	19 (3.8%)	57986
5	58 (3.2%)	97 (3.5%)	24 (4.8%)	161227
6	20 (1.1%)	40 (1.4%)	4 (0.8%)	38565
7	4 (0.2%)	4 (0.1%)	1 (0.2%)	17969

Western Australia

**MMM2019**	**Profession**			**Usual resident Population**
	**Occupational Therapist (n=3232)**	**Physiotherapist (n=4030)**	**Podiatrist (n=505)**	
1	2797 (86.5%)	3537 (87.8%)	434 (85.9%)	1916626
2	95 (2.9%)	112 (2.8%)	14 (2.8%)	110088
3	173 (5.4%)	187 (4.6%)	33 (6.5%)	137241
4	25 (0.8%)	42 (1.0%)	5 (1.0%)	24625
5	58 (1.8%)	61 (1.5%)	6 (1.2%)	127620
6	62 (1.9%)	64 (1.6%)	12 (2.4%)	83974
7	22 (0.7%)	27 (0.7%)	1 (0.2%)	67718

Tasmania

**MMM2019**	**Profession**			**Usual resident Population**
	**Occupational Therapist (n=338)**	**Physiotherapist (n=537)**	**Podiatrist (n=115)**	
2	281 (83.1%)	429 (79.9%)	88 (76.5%)	322658
3	45 (13.3%)	77 (14.3%)	25 (21.7%)	79518
4	0	0	0	3530
5	11 (3.3%)	27 (5.0%)	2 (1.7%)	92248
6	1 (0.3%)	3 (0.6%)	0 (0.0%)	8485
7	0 (0.0%)	1 (0.2%)	0 (0.0%)	2487

Northern Territory

**MMM2019**	**Profession**			**Usual resident Population**
	**Occupational Therapist (n=194)**	**Physiotherapist (n=204)**	**Podiatrist (n=26)**	
2	152 (78.4%)	150 (73.5%)	15 (57.7%)	133648
5	1 (0.5%)	0 (0.0%)	0 (0.0%)	3230

6	35 (18.0%)	44 (21.6%)	9 (34.6%)	43056
7	6 (3.1%)	10 (4.9%)	2 (7.7%)	46392

Australian Capital Territory

MMM2019	Profession			Usual resident Population
	**Occupational Therapist (n= 367)**	**Physiotherapist (n=670)**	**Podiatrist (n=66)**	
1	367 (100.0%)	664 (99.1%)	63 (95.5%)	396218
2	0 (0.0%)	6 (0.9%)	3 (4.5%)	623
3	0	0	0	42
