# Supplementary material for: The distribution of registered occupational therapists, physiotherapists, and podiatrists in Australia
Source: PLoS One. 2023 Sep 21;18(9):e0291962. doi: 10.1371/journal.pone.0291962 (PMC10513188; doi:10.1371/journal.pone.0291962)
Supplement: S2 Table — (PDF) [file pone.0291962.s002.pdf]

S2 Table. Proportion of registered occupational therapists, physiotherapists, and podiatrists by SA2 IRSAD quintiles in Australia, April 2020

__NB__: 313 professionals were excluded from the analysis because some SA2 areas do not have IRSAD scores

Australia

SA2_IRSAD quintile	Profession			Usual resident Population
	Occupational Therapist (n=23100)	Physiotherapist (n=33686)	Podiatrist (n=5489)	
1	3020 (13.1%)	3639 (10.8%)	776 (14.1%)	4107313
2	3694 (16.0%)	4468 (13.3%)	960 (17.5%)	4456655
3	4328 (18.7%)	5666 (16.8%)	1048 (19.1%)	4859413
4	5500 (23.8%)	8115 (24.1%)	1208 (22.0%)	4859489
5	6558 (28.4%)	11798 (35.0%)	1497 (27.3%)	5069035

__NB:__ The total population excludes residents living in SA2 areas (126 SA2 areas) that did not receive IRSAD score (n= 49,992).

New South Wales

SA2_IRSAD quintile	Profession			Usual resident Population
	Occupational Therapist (n=6524)	Physiotherapist (n=10191)	Podiatrist (n=1542)	
1	916 (14.0%)	1187 (11.6%)	222 (14.4%)	1296662
2	1238 (19.0%)	1537 (15.1%)	344 (22.3%)	1559299
3	1177 (18.0%)	1610 (15.8%)	256 (16.6%)	1210215
4	1345 (20.6%)	1992 (19.5%)	298 (19.3%)	1329722
5	1848 (28.3%)	3865 (37.9%)	422 (27.4%)	2071311

Victoria

SA2_IRSAD quintile	Profession			Usual resident Population
	Occupational Therapist (n=6046)	Physiotherapist (n=8716)	Podiatrist (n=1762)	
1	794 (13.1%)	838 (9.6%)	218 (12.4%)	815330
2	1057 (17.5%)	1272 (14.6%)	327 (18.6%)	1153123
3	1014 (16.8%)	1197 (13.7%)	323 (18.3%)	1220990
4	1297 (21.5%)	1834 (21.0%)	358 (20.3%)	1340590
5	1884 (31.2%)	3575 (41.0%)	536 (30.4%)	1388987

Queensland

SA2_IRSAD quintile	Profession			Usual resident Population
	Occupational Therapist (n=4706)	Physiotherapist (n=6675)	Podiatrist (n=981)	
1	680 (14.4%)	869 (13.0%)	156 (15.9%)	1023879
2	743 (15.8%)	795 (11.9%)	117 (11.9%)	804956
3	1110 (23.6%)	1573 (23.6%)	267 (27.2%)	1198731
4	1189 (25.3%)	1916 (28.7%)	238 (24.3%)	1058999
5	984 (20.9%)	1522 (22.8%)	203 (20.7%)	603814

South Australia

SA2_IRSAD quintile	Profession			Usual resident Population
	Occupational Therapist (n=1805)	Physiotherapist (n=2752)	Podiatrist (n=503)	
1	324 (18.0%)	372 (13.5%)	96 (19.1%)	437953
2	315 (17.5%)	463 (16.8%)	110 (21.9%)	445624
3	394 (21.8%)	516 (18.8%)	80 (15.9%)	373717
4	468 (25.9%)	862 (31.3%)	137 (27.2%)	247597
5	304 (16.8%)	539 (19.6%)	80 (15.9%)	168710

Western Australia

SA2_IRSAD quintile	Profession			Usual resident Population
	Occupational Therapist (n=3133)	Physiotherapist (n=3957)	Podiatrist (n=497)	
1	222 (7.1%)	251 (6.3%)	44 (8.9%)	243354
2	316 (10.1%)	354 (8.9%)	58 (11.7%)	381931
3	527 (16.8%)	628 (15.9%)	98 (19.7%)	700122
4	857 (27.4%)	1028 (26.0%)	97 (19.5%)	621906
5	1211 (38.7%)	1696 (42.9%)	200 (40.2%)	520483

Tasmania

SA2_IRSAD quintile	Profession			Usual resident Population
	Occupational Therapist (n=338)	Physiotherapist (n=537)	Podiatrist (n=115)	
1	76 (22.5%)	118 (22.0%)	38 (33.0%)	230291
2	23 (6.8%)	45 (8.4%)	4 (3.5%)	103074
3	54 (16.0%)	81 (15.1%)	18 (15.7%)	85270
4	176 (52.1%)	279 (52.0%)	50 (43.5%)	70341
5	9 (2.7%)	14 (2.6%)	5 (4.3%)	20003

Northern Territory

SA2_IRSAD quintile	Profession			Usual resident Population
	Occupational Therapist (n=194)	Physiotherapist (n=204)	Podiatrist (n=26)	
1	6 (3.1%)	4 (2.0%)	2 (7.7%)	58069
2	2 (1.0%)	1 (0.5%)	0 (0.0%)	5669
3	45 (23.2%)	52 (25.5%)	5 (19.2%)	59152
4	93 (47.9%)	95 (46.6%)	11 (42.3%)	56991
5	48 (24.7%)	52 (25.5%)	8 (30.8%)	46325

Australian Capital Territory

SA2_IRSAD quintile	Profession			Usual resident Population
	Occupational Therapist (n=354)	Physiotherapist (n=654)	Podiatrist (n=63)	
1	2 (0.6%)	0 (0.0%)	0 (0.0%)	840
2	0 (0.0%)	1 (0.2%)	0 (0.0%)	2979
3	7 (2.0%)	9 (1.4%)	1 (1.6%)	7625

4	75 (21.2%)	109 (16.7%)	19 (30.2%)	133343
5	270 (76.3%)	535 (81.8%)	43 (68.3%)	249402
