# Supplementary material for: The distribution of registered occupational therapists, physiotherapists, and podiatrists in Australia
Source: PLoS One. 2023 Sep 21;18(9):e0291962. doi: 10.1371/journal.pone.0291962 (PMC10513188; doi:10.1371/journal.pone.0291962)
Supplement: Attachment — Submitted filename: Response to Reviewers.docx [file pone.0291962.s003.docx]

**"Geographic distribution of registered occupational therapists, physiotherapists and podiatrists in Australia"** (**MS**: PONE-D-22-18539).

Dear Editor,

We are very grateful for the constructive comments from the editor and reviewers. We have taken due consideration of the comments provided and made the necessary revisions to the manuscript. All page and line numbers refer to the marked copy of the manuscript.

**Response to** **Editorial comments**

**COMMENT:** Please ensure that your manuscript meets PLOS ONE's style requirements, including those for file naming.

**AUTHORS’ RESPONSE**: In the revision, we have re-formatted our manuscript according to the PLOS ONE’s style, including the file naming.

**COMMENT:** Please provide additional details regarding participant consent. In the ethics statement in the Methods and online submission information, please ensure that you have specified (1) whether consent was informed and (2) what type you obtained (for instance, written or verbal, and if verbal, how it was documented and witnessed). If your study included minors, state whether you obtained consent from parents or guardians. If the need for consent was waived by the ethics committee, please include this information.

If you are reporting a retrospective study of medical records or archived samples, please ensure that you have discussed whether all data were fully anonymized before you accessed them and/or whether the IRB or ethics committee waived the requirement for informed consent. If patients provided informed written consent to have data from their medical records used in research, please include this information

Once you have amended this/these statement(s) in the Methods section of the manuscript, please add the same text to the “Ethics Statement” field of the submission form (via “Edit Submission”).

For additional information about PLOS ONE ethical requirements for human subjects research, please refer to http://journals.plos.org/plosone/s/submission-guidelines#loc-human-subjects-research.

**AUTHORS’ RESPONSE**: Given that we have utilised publicly available data sources, ethics approval and the need for participant consent for this study were exempted by the University of South Australia’s Human Research Ethics Committee (Application ID: 203544). In the revision, we have stated this in the methods section of our manuscript, and we have added the same text to the “Ethics Statement” field of the submission form. Our revision can be found in the methods section track changed.

**Response to** **Reviewer #1**

**COMMENT:** This is a relatively simple paper that describes the distribution of Australia’s allied health workforce (physio, OT, podiatry) at both a state and national scale, against 2 classifications measuring (1) rurality; (2) socio-economic status / IRSAD. There are many improvements required to its presentation.

**AUTHORS’ RESPONSE**: In the revision, we have significantly improved the presentation of results and interpretation based on the reviewers’ comments. Our revision can be accessed in the track changes in the revised manuscript.

**COMMENT:** Their title is misleading – they state, “geographic distribution”, but IRSAD is technically not a geographical scale. It is a demographic classification – knowing that there are more services in a high IRSAD area does not by itself reveal geog distribution, without adding an extra geographic layer of where the high IRSAD areas are located.

**AUTHORS’ RESPONSE**: We apologise for our misleading title. We have removed the term ‘geographic’ from our title and have revised the title as “*The distribution of registered occupational therapists, physiotherapists and podiatrists in Australia*". We appreciate that although IRSAD measures the socioeconomic status of an area, it is a sociodemographic measure and may not be used to describe geographic classification on its own. We have revised the text of our manuscript accordingly and our revision can be found in track changes in the revised manuscript.

**COMMENT:** I’m also not comfortable with their reference to the MM classification as a measure of “geographic access” – it is not. I am aware of reference #11 describing it in this way, but in this paper, it is actually quite confusing and misleading to use this phrase. The one ‘access’ measure this paper does utilise is the provider-to-population ratio (i.e., ‘density’), but confusingly the authors are not referring to that. The MM classification is a measure of ‘rurality’, it combines a geographic component (remoteness) with a demographic component (population size). The authors may be aware that the original measure of ‘remoteness’ (ARIA) had the word ‘accessibility’ in its name and some of the category labels used ‘accessible’; however, this was highly criticised because it was NOT a measure of access, hence it was changed to the newer RA scale and variants of the word ‘access’ were wholly removed. The MM scale (which is partly defined by the RA scale) should also be applied this way.

**AUTHORS’ RESPONSE**: Thank you for the comments. We agree that the MMM classification should not be used as a measure of “geographic access”. We have removed the phrase “geographic access” and replaced it with the appropriate phrase throughout the manuscript. We have also focused on the ‘Provider-to-population ratio (i.e., ‘density’)’ as a measure of access as per your suggestion in the revised manuscript. Our revision can be found as track changes in the revised manuscript.

**COMMENT:** I was somewhat disappointed by their presentation / discussion of the results. The authors almost wholly focus on the extreme categories only – those with the highest workforce density (can call this ‘access’) and those with the least. Unsurprisingly, this largely matches the extreme categories of both scales. The authors largely ignore whatever patterns are observed within the middle categories, which is arguably where much of the interest of these data is.

**AUTHORS’ RESPONSE**: Thank you for this comment. In the revision, we have considered all categories of the MMM and IRSAD classification when presenting and discussing the results in our revised manuscript. In the whole of Australia, the highest density of the three allied health workforce was found in areas classified as MM 1 and decreased with the increasing levels of the MMM categories. Furthermore, in the whole of Australia, the highest density of the three allied health workforce was found in areas with IRSAD quintile 5 and decreased with the declining levels of IRSAD quintiles. However, the pattern in the distribution of the three allied health workforce densities varied widely by the MMM categories and IRSAD quintiles across each state and territory. Our revision can be accessed in track changes in the “Results” and “Discussion” sections.

**COMMENT:** I think that part of the problem is that the authors have too many layers they’re trying to describe, forgetting that ‘distribution’ is the primary interest. Having 3x clinician types means that there are three patterns to describe, with little gained from if they focused on one. Jurisdictional differences may be important to some service planners, but generally add little to the paper’s value. Moreover, having 2x distribution scales doubles the observed patterns. Specific distributional differences should be the majority point of focus (e.g. what is the step down/up ratio of density (access) between categories of the MM / IRSAD classifications? Why is MM-6 generally ‘better’ than MM-5 / MM-5? (PS: this is not surprising; the MM scale is not meant to always be ordinal).

**AUTHORS’ RESPONSE**: In the revision, we have focused on describing the distributional differences of the three allied health workforce densities stratified by the MMM and IRSAD in the whole of Australia as well as in each state and territory as per your comments. Our revision can be found in track changes in the text of the revised manuscript.

**COMMENT**: Where required, the authors don’t clarify certain patterns – chief example is the highest density in NT and Tas being in MM-2, rather than MM-1 (which is 100% explained by there being no MM-1 locations in either jurisdiction). Also, the authors at no point discuss the inherent association(s) between the MM and IRSAD scales. IRSAD differences (particularly at the higher end) will likely mostly be determined by metropolitan locations (ie. within MM-1). The last sentence prior to the Strengths directly assumes assoc between lower density and lower IRSAD relates to rural communities, but they have never actually demonstrated a linkage between MM and IRSAD.

**AUTHORS’ RESPONSE**: Thank you for this comment. In the revision, we have added sentences to the revised manuscript to explain the distributional patterns observed in the Northern Territory and Tasmania as per your suggestion (see page 19, lines 334-336). We have also added a couple of new sentences to the revised manuscript to explain the inherent association between the MMM and IRSAD classifications in the revised manuscript (see page 21, lines, 368-373).

**COMMENT**: Reflecting on my earlier point, the first sentence of the conclusion is nonsensical – ‘access’ belongs with the uneven distribution part of the sentence. The next sentence is not appropriate (‘accessible’ and ‘least accessible’ are incorrect labels). The last sentence is OK, with ‘equity of access’ being about the service levels (density). The Abstract conclusion needs to be similarly fixed up.

**AUTHORS’ RESPONSE**: We have revised the ‘Conclusion’ section as per your comments, and we now believe that the conclusion is appropriate. We have also revised the conclusion in the Abstract. Our revision can be found in track changes in the revised manuscript.

**COMMENT:** Another nonsensical statement is at lines 257-259 (“Conversely, the lower densities…”), which essentially is saying the ‘lower access (density)’…may explain the ‘lower access (availability)’! Following this, the heading “Variation by access and jurisdiction” needs changing.

**AUTHORS’ RESPONSE**: We have revised the stated sentence to make the message being conveyed clearer. We have also revised the heading that reads, “Variation by access and jurisdiction” as “Variation in the density of the three allied health workforce by an objective measure of rurality and jurisdiction”. Our revision can be accessed in track changes in the revised manuscript.

**COMMENT:** One paragraph didn’t make sense to me – starting at line 189; it twice refers to NSW, SA, Tas, NT, ACT but I can’t determine why they are stated twice.

**AUTHORS’ RESPONSE**: In the revision, we have grossly revised the stated paragraph to make the message conveyed clearer.

**Response to** **Reviewer #2**

**Major comments**

**COMMENT:** While this may be the first study of this kind in Australia, this type of study has been done in other countries including USA and Canada. The authors are encouraged to expand introductions and discussions comparing their findings with other studies. Are there any novel findings from this particular study?

**AUTHORS’ RESPONSE**: We understand that there are a few studies conducted in the USA and Canada regarding the distribution of the allied health workforce, with a focus on physiotherapists and occupational therapists. In the revision, we have added some more new references to expand the ‘Introduction’ and ‘Discussion’ sections as per your comments. Our revision can be found in track changes in the revised manuscript.

Given that our current study is the first study in Australia that described the distribution of three allied health workforce (physiotherapists, occupational therapists, and podiatrists) according to an objective measure of location/rurality and area-level socioeconomic status, there are several novel findings from this study. These include: (1) In the whole of Australia, the highest densities of the three allied health workforce were found in areas classified as MM 1 and decreased with the increasing levels of the MMM categories. (2) In the whole of Australia, the highest densities of the three allied health workforce were found in areas with IRSAD quintile 5 and decreased with the declining levels of IRSAD quintiles. (3) The patterns of the three allied health workforce densities were unevenly distributed by MM categories and IRSAD quintiles across each state and territory. (4) The densities of these three groups of allied health workforce appeared to be more concentrated in areas classified from MM 1-4 across each state and territory while areas classified from MM 5-7 exhibited less allied health workforce density.

**Minor comments**

**COMMENT:** Abstract: The authors are requested to include the study objective after the background.

**AUTHORS’ RESPONSE**: In the revision, we have included the study objective in the Abstract. Our revision can be accessed in track changes in the revised manuscript.

**COMMENT:** The background needs to describe in more detail why this study is so critical?

**AUTHORS’ RESPONSE**: In the revision, we have added more descriptions regarding why our current study is very important. Our revision can be found in the track changes in the revised manuscript (see page 8, lines, 128-132).

**COMMENT:** Discussion section also did not present according to the research purpose and result. The authors are encouraged to Interpret and explain study results, and critically compare to previous studies. For example, at first occupational therapist then physiotherapist and finally podiatrist.

**AUTHORS’ RESPONSE**: In the revision, we have added some new explanations and interpretations of the results of our study in the “Discussion” section. We have also re-structured the discussion to align with the study purpose and results. Moreover, we have compared our findings to previous studies conducted in Canada and South Africa. Our revision can be found in the track changes in the revised manuscript.

**COMMENT:** Page 17, line 316-317. This sentence is not a limitation as the authors are focusing their objective based on three professions- occupational therapist, physiotherapist and podiatrist. Please drop it.

**AUTHORS’ RESPONSE**: Thank you for alerting us to this. We have revised it accordingly.
