# Supplementary material for: The distribution of registered occupational therapists, physiotherapists, and podiatrists in Australia
Source: PLoS One. 2023 Sep 21;18(9):e0291962. doi: 10.1371/journal.pone.0291962 (PMC10513188; doi:10.1371/journal.pone.0291962)
Supplement: Attachment — Submitted filename: Response to Reviewers.docx [file pone.0291962.s004.docx]

**"The distribution of registered occupational therapists, physiotherapists and podiatrists in Australia"** (**MS**: PONE-D-22-18539R1).

Dear Editor,

We are very grateful for the constructive comments from reviewers. We have taken due consideration of the comments provided and made the necessary revisions to the manuscript. All page and line numbers refer to the marked copy of the manuscript.

**Response to** **Reviewer #3**

**COMMENT:** This is a descriptive study that presents important information about workforce distribution in Australia. The finding that the allied health workforce is lower per population in more remote regions and those with higher levels of disadvantage is not new, but the detail provided about these three disciplines offers new information. It is important for those involved in workforce planning, development and research to have this information. The paper is also clear, refers to relevant literature and describes the sources of data well. The patterns are described clearly and the implications of these are discussed in the latter sections.

**AUTHORS’ RESPONSE**: Thank you.

**COMMENT:** The study would benefit from being more analytical. More detail about specific regions and the patterns and variations of regions would add to this paper. For example, the authors might provide examples of particular places/regions in different states to give more depth. Is it possible to consider how remoteness and disadvantage overlap (and where they do not)?

**AUTHORS’ RESPONSE**: In the revision, we have added more details regarding the distribution of allied health workforce in specific regions/areas (such as MM 2 regions) across each state and territory in Australia. Our revision can be found in track changes in the revised manuscript (see page 11 and page 13).

Yes, it is possible to consider how remoteness and disadvantage overlap and where they do not. Remoteness and disadvantage can often overlap in areas where access to resources and services is limited due to geographical barriers or economic and social barriers. However, there may be situations where remoteness and disadvantage do not overlap. For instance, individuals living in a remote area may have access to the same resources and services as individuals living in metropolitan area due to availability of infrastructure and technology. Conversely, individuals living in a metropolitan area may experience disadvantage due to poverty, discrimination, or other social and economic factors. In the revision, we have included an explanation highlighting the significance of considering specific context and relevant factors for a thorough analysis of the overlap between remoteness and disadvantage. Our revision can be found tracked changed in the revised manuscript (see page18).

**COMMENT:** There are some minor changes for the authors to consider. First, in the results section of the abstract, it states: “However, there was no clear pattern in the distribution of occupational therapists, physiotherapists, and podiatrists density when stratified by the MMM classifications and IRSAD quintiles across each state and territory in Australia.” I could not find evidence of this in the paper; were MM and IRSAD analysed together? Or is this a general sentence, and if so, I would question whether these was not a pattern across MM regions.

**AUTHORS’ RESPONSE**: The MMM and IRSAD data were analyzed independently. In the revision, we have made improvements to the stated sentence to enhance clarity. The revised sentence can be found in the track changes in the “Abstract” section the revised manuscript.

**COMMENT:** Second, I would have liked a sentence in the limitations section to acknowledge variations and differences within states and within these categories. The macro view is important but rural areas and types of disadvantages differ and it is important to acknowledge this. It is easier to recruit a physio to some rural regions than others.

**AUTHORS’ RESPONSE**: Thank you for your feedback. In the revision, we have acknowledged that further studies should explore the significant disparities in the allied health workforce distribution in Australia when classified by MMM categories and IRSAD quintile in detail across rural and disadvantaged areas. This is essential because some rural areas and disadvantaged populations may have unique characteristics that require targeted solutions. Our revision can be found in track changes in the revised manuscript (page 19 and page 20).

**COMMENT:** Third, on Line 188, it states “…because SA2 represents communities that interact together socially and economically;” is this correct?

**AUTHORS’ RESPONSE**: Yes, it is correct. According to the Australian Bureau of Statistics (ABS), SA2s are designed to represents communities that interact together socially and economically. However, we have made some improvements to the wording in order to enhance the clarity of the message being conveyed. Our revision can be found in track changes on page 10.

**COMMENT:** Fourth, I was unsure why MM was referred to as an “objective” measure.

**AUTHORS’ RESPONSE**: The MMM is designed to provide an objective measure of geographic remoteness because it is based on specific criteria and data points that can be quantified and assessed in a consistent manner. However, like any measure, it is important to note that the MMM is not entirely free from subjectivity or limitations. In the revision, for the sake of clarity, we have removed the word "objective" throughout the revised manuscript. Our revision can be found in track changes.

**COMMENT:** Finally, there are a couple of editing issues: (1) The phrase on Line 69 “lived shorter” is awkward wording. (2) The sentence on Line 144 “The AHPRA data were extracted in April 2020, which were de-identified” is also awkward wording.

**AUTHORS’ RESPONSE**: Thank you for the comments. In the revision, we have made some edits to improve the clarity of the conveyed message. Our revision can be found in track changes in the revised manuscript.

**COMMENT:** Overall, while a descriptive paper, the evidence of the variation in distribution of the allied health workforce in Australia according to remoteness and levels of disadvantage is important. The detailed graphs presented in this paper would be very useful and I would likely cite this paper.

**AUTHORS’ RESPONSE**: Thank you.
