# Supplementary material for: The distribution of registered occupational therapists, physiotherapists, and podiatrists in Australia
Source: PLoS One. 2023 Sep 21;18(9):e0291962. doi: 10.1371/journal.pone.0291962 (PMC10513188; doi:10.1371/journal.pone.0291962)
Supplement: Attachment — Submitted filename: Response to Reviewers.docx [file pone.0291962.s005.docx]

**"The distribution of registered occupational therapists, physiotherapists and podiatrists in Australia"** (**MS**: PONE-D-22-18539R2).

Dear Editor,

We are very grateful for the constructive comments from reviewers. We have taken due consideration of the comments provided and made the necessary revisions to the manuscript. All page and line numbers refer to the marked copy of the manuscript.

**Response to** **Reviewer #5**

**COMMENT:** INTRODUCTION: Abstract: The title of the study and the objective of the study is not matching (include workforce) Please revise it.

**AUTHORS’ RESPONSE**: In the revision, we have incorporated the term "workforce" into the objective stated in the Abstract. Our revision can be found in track changes in the revised manuscript (Page 2).

**COMMENT:** The WHO, rehabilitation group, is being very active in the latter years with activities for strengthening rehab in health systems, with workforce as one of the pillars, with plenty of works, gatherings and citations you could/should use - the most significant. Global Forum on Human Resources for Health, an analysis of the WHO Global Health Observatory Data Repository containing information from 36 countries showed that maintaining a sufficient health care workforce is a global priority and that the effectiveness of that workforce should be determined by calculating the healthcare workforce-to-population ratio. Include these references.

It is importantly to systematically search/approach/use/build over the strengths and gaps of the literature on the topic beforehand.

1. Campbell J, Dussault G, Buchan J, Pozo-Martin F, Guerra Arias M, Leone C, Siyam A, Cometto G. A universal truth: No health without a workforce. Forum Report, Third Global Forum on Human Resources for Health, Recife, Brazil. Geneva, Global Health Workforce Alliance and World Health Organization. 2013. http://www.who.int/workforcealliance/knowledge/ resources/hrhreport2013/en/. Accessed 13 Feb 2017.
2. World Health Organization. Dublin Declaration on Human Resources for Health. Fourth Global Forum on Human Resources for Health. 2017. http://www.who.int/hrh/events/Dublin_Declaration-on-HumanResou rces-for-Health.pdf?ua=1.
3. World Health Organization. Global strategy for human resources for health: workforce 2030. Draft for the 69th World Health Assembly 2016. http://www.who.int/hrh/resources/16059_Global_strategyWorkfor ce2030.pdf?ua=1.

I list at least 3 relevant articles (directly focused on the physiotherapy workforce), that were not cited:

Jesus TS, Koh G, Landry M, et al. Finding the "Right-Size" Physical Therapy Workforce: International Perspective Across 4 Countries. Physical therapy 2016;96(10):1597-609. doi: 10.2522/ptj.20160014 [published Online First: 2016/05/07]

Landry MD, Hack LM, Coulson E, et al. Workforce Projections 2010-2020: Annual Supply and Demand Forecasting Models for Physical Therapists Across the United States. Physical therapy 2016;96(1):71-80. doi: 10.2522/ptj.20150010 [published Online First: 2015/10/17]

Shah TI, Milosavljevic S, Trask C, et al. Mapping Physiotherapy Use in Canada in Relation to Physiotherapist Distribution. Physiotherapy Canada Physiotherapie Canada 2019;71(3):213-19. doi: 10.3138/ptc-2018-0023 [published Online First: 2019/11/14]

**AUTHORS’ RESPONSE**: Thank you for suggesting these references. In the revised manuscript, we have incorporated citations for most of the mentioned references (see Page 4).

**COMMENT:** What’s new in the scientific literature with this manuscript? Include in introduction.

**AUTHORS’ RESPONSE**: While previous studies have explored aspects of health workforce distribution, few have comprehensively analysed the allied health workforce's density on a national and state/territory level, considering both socioeconomic characteristics and geographical location. Our manuscript uniquely aimed that combining the measures of location/rurality and area-level socioeconomic position as well as the total resident population to provide a new insight into the complex interplay between health workforce availability, socioeconomic factors, and geographic disparities. This approach is important to deliver policy relevant insights aimed at promoting equitable access to allied health workforce. In the revision, we have incorporated what our manuscript adds to the scientific literature in the Introduction section. Our revision can be found in track changes in the revised manuscript (see Page 7).

**COMMENT:** The manuscript must be include a hypothesis. Explain the hypothesis.

**AUTHORS’ RESPONSE**: Given that our study is a descriptive quantitative study, our primary focus is on summarizing and presenting data based on our research objective/question rather than testing a specific hypothesis. However, we have provided/added a clear description of our research objective that can serve as a guiding framework for our study (see Introduction page 7).

**COMMENT**: METHODS: "We linked data from three sources: (1) the public registration data of occupational therapists, physiotherapists, and podiatrists obtained from the Australian Health Practitioner Regulation Agency (AHPRA), the Modified Monash Model (MMM) 2019 data from the Australian Department of Health; and the Socio-Economic Indexes for Areas (SEIFA)”- Why are 3 data sources needed? Explain. Do all these agencies have committees in place to study the markers of an effective workforce, to manage demands related to access, and to create and implement public policies aimed at improving their health systems?

**AUTHORS’ RESPONSE**: We used the three data sources (by linking each of them) to explore the interplay between allied health workforce availability, measure of location/rurality, and socioeconomic position. This approach is important to deliver policy relevant insights aimed at promoting equitable healthcare access and outcomes. The first data source, the AHPRA, plays a foundational role by providing information about registered professionals. This dataset includes details about occupational therapists, physiotherapists, and podiatrists, providing valuable insights into their numbers and locations. The second source, the MMM 2019 data, offers a geographic classification system that categorises areas based on their remoteness and population size. The third source, the SEIFA, delves into socio-economic factors at different geographic levels, offering insights into the relative socio-economic advantage or disadvantage of different areas. By incorporating SEIFA data, we gain a deeper understanding of the socio-economic context within which healthcare practitioners operate.

Regarding the presence of committees within these agencies for studying workforce markers, managing access demands, and creating/implementing public policies:

- The AHPRA is primarily responsible for the registration and regulation of health practitioners. While AHPRA itself might not have committees specifically focused on studying workforce markers or creating public policies, it collaborates with various professional boards (e.g., Physiotherapy Board, Occupational Therapy Board) to ensure practitioner standards and regulatory measures are in place.
- The Australian Department of Health, which provides the Modified Monash Model (MMM) data, may have committees or departments dedicated to healthcare workforce planning, policy development, and access management. These committees might analyze data trends to ensure that healthcare services are appropriately distributed, especially in underserved areas.
- SEIFA is a product of the ABS. The ABS might not have specific committees focused on healthcare, but the SEIFA data is widely used by various government agencies and researchers to understand socio-economic disparities and inform policy decisions, including those related to healthcare.

Therefore, while these agencies may not have dedicated committees solely for healthcare workforce markers, access demands, and policy creation, they are likely involved in these areas through collaboration, data provision, and policy implementation within their broader mandates. In the revision, we provide a clear description of why these data sources are need in our study. Our revision can be found in track changes (see Page 8.)

**COMMENT**: DISCUSSION: Include the strengths of the study.

**AUTHORS’ RESPONSE:** We have already included the strength of our study in the 'Discussion' section (see page 19).

**COMMENT**: The final paragraph should leave the reader with your final message within the framework of the hypotheses posed in the Introduction.

**AUTHORS’ RESPONSE**: In the Conclusion section, we have highlighted the notable disparity in the distribution of registered occupational therapists, physiotherapists, and podiatrists when categorised by measures of location/rurality and area-level socioeconomic status across Australian jurisdictions. These findings align with the research question/objective outlined in our Introduction, which focused on describing the density of these three groups of allied health workforce based on location/rurality and area-level socioeconomic status across Australian jurisdictions. The pronounced geographic imbalances in access to allied health workforce underscore the need for targeted policy interventions and comprehensive workforce planning. These measures are crucial in ensuring that healthcare services remain accessible and equitable for all Australians, irrespective of their geographic location or socioeconomic status. In the revision, we have added some more explanation in the last paragraph to enhance the clarity of our final message. Our revision can be found in track changes (see Page 21).

**COMMENT**: Include the clinical significance of this study over clinicians, patients, and researchers after the study hypothesis.

**AUTHORS’ RESPONSE**: In the revision, we have added some discussion regarding the significance of our findings. Our revision can be accessed track changed in the ‘Discussion’ section in the revised manuscript (see Page 19).

**Response to** **Reviewer #6**

**COMMENT**: You may remove "the latest available data" on Line 79 as it appears redundant.

**AUTHORS’ RESPONSE**: Thank you. We have revised it as per the suggestion.

**COMMENT**: Also, the text in lines 327-329 appears redundant as the point was made clear in the lines 287-289. However, if it was restated for emphasis, please ignore this suggestion.

**AUTHORS’ RESPONSE**: Thank you for pointing this. We have restated the stated text for emphasis and explanation.
